# **C** **omparison between DaVinci** ^®^ **and Hugo** ^**™**^ **-RAS Roux-en-Y Gastric Bypass in bariatric surgery**

**DOI:** 10.1007/s11701-024-02063-w

**Published:** 2024-08-06

**Authors:** Francesco Pennestrì, Giuseppe Marincola, Priscilla Francesca Procopio, Pierpaolo Gallucci, Giulia Salvi, Luigi Ciccoritti, Francesco Greco, Marco Raffaelli

**Affiliations:** 1 U.O.C. Chirurgia Endocrina e Metabolica, Fondazione Policlinico Universitario Agostino Gemelli IRCCS, Rome, Italy; 2 Centro di Ricerca di Chirurgia delle Ghiandole Endocrine e dell’Obesità, Università Cattolica del Sacro Cuore, Rome, Italy

**Keywords:** Hugo^™^-RAS, DaVinci^®^, Roux-en-Y Gastric Bypass, Severe obesity, Complications, Outcomes

## Abstract

The application of a robotic platform in the bariatric surgical field is intended to enhance the already established advantages of minimally invasive surgery in terms of both technical and clinical outcomes. These advantages are especially relevant for technically challenging multiquadrant operations such as Roux-en-Y Gastric Bypass (RYGB). Consequently, robotic-assisted surgery has emerged as a possible application for bariatric surgeries. The study attempts to assess feasibility and safety of the Hugo^™^-Robotic-Assisted Surgery System (Hugo^™^-RAS) platform compared to the DaVinci^®^ Surgical System (DaVinci^®^-SS) with a focus on complication rates and operative times. We retrospectively reviewed bariatric procedures performed from January 2013 until December 2023. We included all robotic RYGBs performed using Hugo^™^-RAS and DaVinci^®^-SS platforms. The study utilized Propensity Score Matching (PSM) analysis to address bias in selection, matching patients based on age, gender, body mass index, comorbidities and past abdominal operations. One hundred thirty-five patients were identified: 90 DaVinci^®^-SS and 45 Hugo^™^-RAS procedures. After PSM, each group consisted of 45 patients. There was no discernible disparity observed in relation to early (≤ 30 days) postoperative complications rate (*p* = 1), mean operative time (for docking time, console time and total operative time: *p* = 0.176, *p* = 0.678, *p* = 0.229, respectively) and postoperative hospital stay (*p* = 0.052) between DaVinci^®^-RSS and Hugo^™^-RAS procedures. Our results suggest that application of both Hugo^™^-RAS and DaVinci^®^-SS robotic platforms to RYGB is related to comparable safety profiles. Although DaVinci^®^-SS remains the most widely adopted platform in clinical practice, this study underscores the potential role of the Hugo^™^-RAS to provide effective solutions in robotic bariatric procedures.

**Supplementary Information:**

The online version contains supplementary material available at 10.1007/s11701-024-02063-w.

## Introduction

The application of robotic platforms to bariatric operations aims to further improve the technical advantages of minimally invasive surgical procedures regarding precision, ergonomics and efficiency [1].

This is particularly applicable to multiquadrant interventions involving reconstructive phases, including Roux-en-Y Gastric Bypass (RYGB), which is a frequently practiced type of bariatric surgical interventions [2, 3]. Indeed, the technical challenges due to the realization of hand-sewn anastomoses or running sutures for defects closure led to an increased inclination towards potentially facilitating ergonomic robotic solutions, especially in case of limited working space due to thick abdominal wall and excess abdominal fat [4–7].

Initially described over two decades ago, the first robotic procedure in bariatric surgery set expectations for a new surgical era [8]. Although laparoscopy had a profound effect on bariatric surgery, resulting in a substantial rise in the number of bariatric procedures performed globally, the use of robotic platforms did not yield similar outcomes [9]. Such evidence may be explained by similar complication rates compared to traditional laparoscopic approaches. However, some authors identified specific patients’ cohorts which seem to benefit from robotic surgeries the most, such as Class IV or higher obesity patients and surgery for suboptimal clinical response, suggesting a still valuable role for this technology [1, 7, 10–13].

Nevertheless, the significantly higher costs still represent the strongest drawback against its diffusion [14]. These economic issues are partly due to the longstanding monopoly of the established platform DaVinci^®^ Surgical System (DaVinci^®^-SS—Intuitive Surgical, Sunnyvale, CA, USA) [15]. Hugo^™^-Robotic-Assisted Surgery System (Hugo^™^-RAS—Medtronic, Minneapolis, MN, USA) was introduced in 2022 after gaining “*Conformité Européenne*” certification for clinical practice [16–19]. Thereafter, Hugo^™^-RAS application to bariatric surgery has been described [20] and its preliminary results have shown feasibility and reproducibility [21]. On the other hand, the introduction of this new robotic platform, equipped with innovative technologies and potential competitive pricing strategies, may lead to a relevant shift in the current economic balance as a contender to the DaVinci^®^-SS system.

To date, DaVinci^®^-SS still represents the standard choice for robotic surgery in clinical practice. However, comparative studies among DaVinci^®^-SS system and other robotic platforms applied to bariatric surgery are currently lacking.

In this retrospective cohort study, we assess the security and efficiency of the Hugo^™^-RAS platform, compared to its established counterpart, with a focus on perioperative complications, operative times (OTs) and postoperative hospital stays (POS).

## Materials and methods

The DaVinci^®^-SS system was initially implemented in our Department in January 2013 [7]. In detail, the Si platform was utilized 2014, when it was displaced by the newer Xi platform. Hugo^™^-RAS was applied to bariatric surgery in our Division from January 2023 [20].

Comprehensive data of bariatric patients have been systematically and prospectively recorded in a dedicated database, which has been rigorously anonymized to ensure patients’ confidentiality.

### Study population

Data of bariatric surgeries performed between January 2013 and December 2023 in our center were registered.

Participants’ selection for the present study followed the agreed-upon criteria for bariatric surgical procedures and complied with the protocols outlined by the nationwide directives of the *SICOb* society (Società Italiana di Chirurgia dell’Obesità e delle malattie metaboliche—Italian Society of Bariatric Surgery and Metabolic Disorders) [22].

All robot-assisted (RA) RYGBs from January 2013 until December 2023 were included in this investigation. Within all the registered minimally invasive primary bariatric surgeries, DaVinci^®^-SS-RYGBs and Hugo^™^-RAS-RYGBs patients who met the specific requirements were assessed for the research using the intention-to-treat analytical framework.

Exclusion criteria consisted in reoperative surgeries, procedures different from RYGB, as well as concurrent surgeries at the time of operation.

In this series, all operations have been performed by senior surgeons with high experience in minimally invasive bariatric procedures. More in detail, all DaVinci^®^-SS-RYGBs were conducted by surgeons who performed a minimum of 200 laparoscopic RYGBs as part of their initial learning process. All Hugo^™^-RAS-RYGBs were conducted by surgeons who undergo a minimum of 30 DaVinci^®^-SS-bariatric operations, as part of their initially robotic learning process.

Study participants were stratified into two cohorts based on the robotic surgical platform used during the procedure: the DaVinci^®^-SS group and the Hugo^™^-RAS group.

The specific surgical platform (DaVinci^®^-SS vs Hugo^™^-RAS) was chosen based on availability of the specific platform on the operation date, surgeons’ preference and patients’ characteristics.

Patients’ baseline and demographics characteristics included sex as assigned at birth, age and Body Mass Index (BMI). Participants’ evaluation encompassed preoperative conditions such as High Blood Pressure, Obstructive Sleep Apnea Syndrome, Diabetes Mellitus Type 2, Non-Alcoholic Fat Liver Disease (NAFLD), as well as prior abdominal surgery. OT and intraoperative complications were also considered. Intensive care unit (ICU) stay, length of POS, early (within 30 days) postoperative complications and readmission rate were evaluated among the postoperative parameters. The follow-up ended on 30 April 2024.

This study adhered to the ethical guidelines according to the Helsinki’s Declaration. We obtained ethical endorsement from our Local Ethical Committee, which conducted a comprehensive revision of the study protocols identified by the numbers 00010895/23. Informed consent was obtained from each participant.

### Study end-points

The primary endpoint of this research was the comparison in terms of the complications rate linked to the DaVinci^®^-SS and the Hugo^™^-RAS platforms. The secondary endpoint was to evaluate and compare the two robotic platforms in terms of OT and POS.

### Definitions

The comprehensive preoperative evaluation for bariatric surgery, involving several medical check-ups, has been previously described in detail [23]. The OT refers to the period starting from the initial incision and ending with the closure of the surgical site, including the connection of the robotic equipment (docking step). The Clavien–Dindo classification was employed to evaluate the intensity of postoperative complications, with observations recorded up to the 30^th^ day after surgery [24].

### Surgical technique

Surgical techniques for RYGB performed either with the DaVinci^®^-SS and the Hugo^™^-RAS platforms have been previously described [7, 20, 21]. However, some technical details must be reported. The surgical steps were the same regardless the chosen robotic platform. In this series all operations were performed by means of laparoscopic staplers. All RYGB surgeries were performed using a double-loop approach. A 40 Fr orogastric bougie was utilized to calibrate the gastric pouch. The surgical intervention was carried out to create the antecolic anastomosis between the gastric pouch and the enterotomy on the jejunal loop, placed 75 cm from the ligament of Treitz. The connection was realized using a 3/0-running barbed sutures. The anastomosis was hand-sewn and consisted of two layers. A jejuno-jejunal anastomosis was made 150 cm away from the gastro-jejunal one by means of a 60 mm laparoscopic stapler. The defect was sealed with a 3/0-running barbed suture.

Patients’ postoperative protocol has been previously described [7, 21, 23].

### Statistical analysis

With the aim of minimizing potential biases in outcomes evaluation, the two groups were carefully selected to have similar characteristics such as sex, age, BMI and comorbidity. This matching process was conducted using propensity score matching (PSM) analysis. PSM was obtained with the “1:1 nearest neighbor” blinded matching method (discard = both groups, caliper = 0.01). Type of robotic platform (Hugo^™^-RAS and DaVinci^®^-SS) was entered into the regression model of PSM as the binary treatment parameter. The following covariates, estimated to be important for the primary outcome, were included into the analysis: sex as assigned at birth (male vs female), age, preoperative BMI and comorbidities. Baseline characteristics, operative and postoperative variables were compared using a bivariate analysis. Normal distribution was evaluated using the means of the Shapiro–Wilk’s test. Fisher’s exact test and Chi-square test were employed to compare categorical variables. Continuous variables were expressed as median (interquartile range, IQR) or mean (± standard deviation, SD). We used paired sample t test or Mann–Whitney U test to compare continuous variables, according to data dispersion of the investigated sample. Basic demographic and medical information were gathered by examining patients’ charts and electronic databases. Statistical analysis and PSM were conducted using Stata version 17.0 (StataCorp, College Station, Texas 77,845 USA). The analyses were conducted using a two-tailed approach, with the probability threshold set at *p* < 0.05.

## Results

**Fig. 1:**
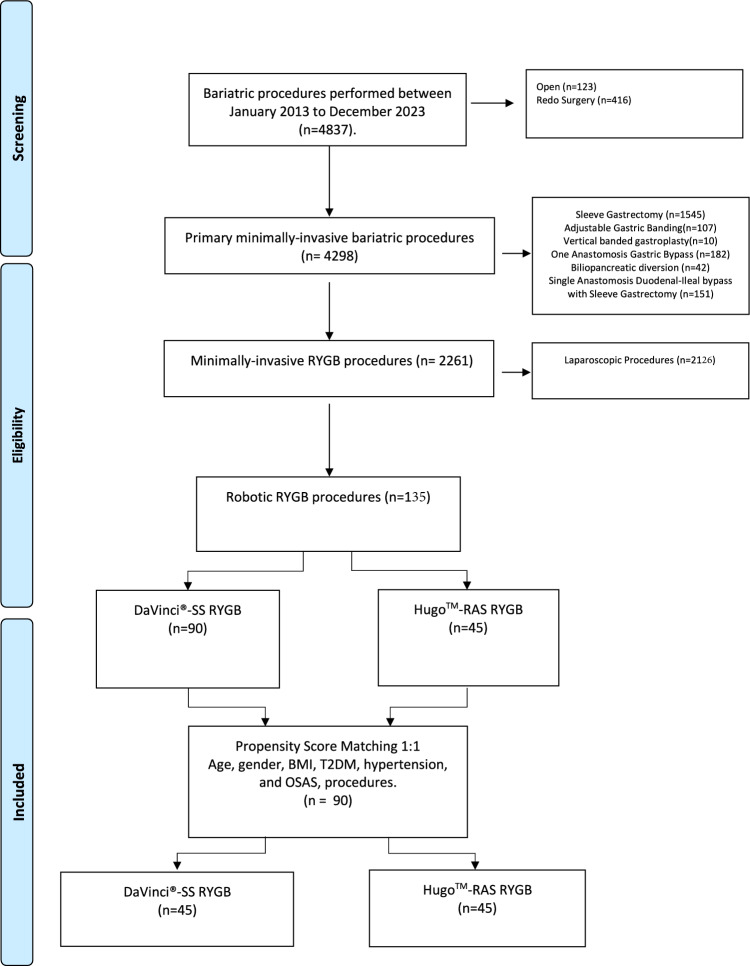
Flowchart illustrating the process of selecting patients for the study

**Fig. 2:**
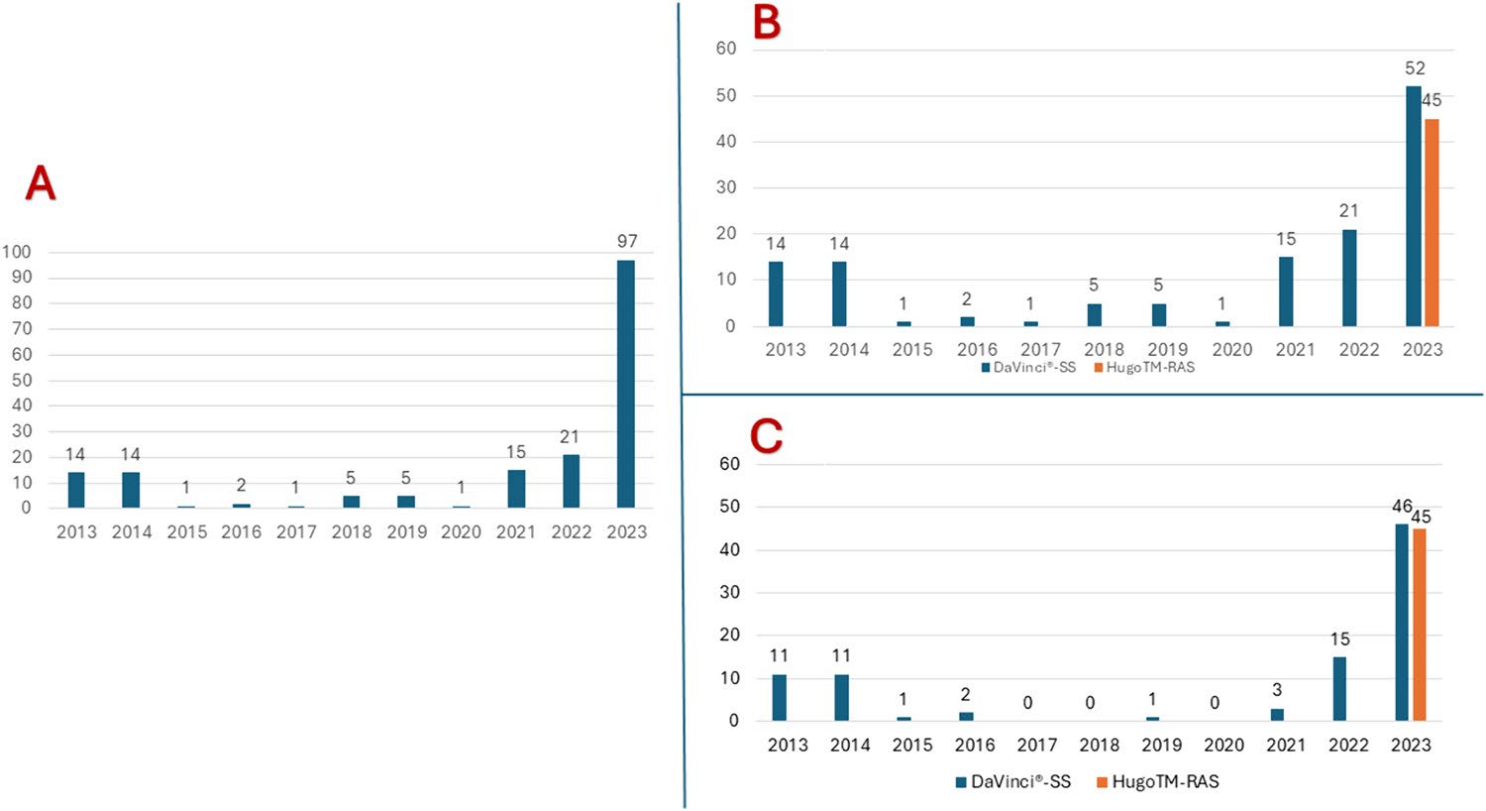
**A** Diagram of robotic procedures per year; **B** Diagram of single platform-robotic procedures per year; **C** Diagram of single platform-Roux-en-Y Gastric Bypass per year

Throughout the study period, an overall number of 4837 patients were designated for bariatric surgery. A total of 4,298 patients received minimally invasive primary bariatric procedures. Out of the entire number of patients, 135 individuals, which accounts for 3.1% of the sample, were eligible to be included in this study (Fig. 1). Figure 2 displays graphs illustrating the robotic procedures conducted throughout the study period.

The comparative investigation in Table 1 examines the clinicopathologic features, intraoperative, and postoperative results of two groups: the Hugo^™^-RAS group consisting of 45 patients and the DaVinci^®^-SS group consisting of 90 patients. Among the evaluated preoperative variables, statistically significant differences were registered in terms of mean BMI (42.1 ± 4.2 kg/m^2^ in the Hugo^™^-RAS group compared to 45.8 ± 6.1 kg/m^2^ in the DaVinci^®^-SS group, *p* = 0.003) and mean weight (121.9 ± 21.1 kg in the Hugo^™^-RAS group, whereas 131.5 ± 24.1 kg in the DaVinci^®^-SS group, *p* = 0.024). There was no notable disparity observed in the distribution of genders and the prevalence of comorbidities (*p* = 0.209 and *p* = 0.163, respectively). The incidence of OSAS statistically differed among the Hugo^™^-RAS and the Da Vinci^®^-SS group (*p* = 0.025). A higher number of DaVinci^®^-SS patients (25.5%) underwent prior laparoscopic surgery compared to 13.3% Hugo^™^-RAS patients; in contrast, the Hugo^™^-RAS group had a higher prevalence of previous open surgery (28.8% vs 6.7% in the DaVinci^®^-RSS group) (*p* = 0.001).

**Table 1:** Comparative analysis of the clinicopathologic characteristics of all patients who met the inclusion criteria: Hugo^™^-RAS RYGB (*n* = 45) vs DaVinci^®^-SS RYGB (*n* = 90)

Variable	Hugo^™^-RAS *N* = 45	DaVinci^®^-SS *N* = 90	*p*-value
Age, years (mean ± SD)	48.1 ± 10.8	43.9 ± 10.4	0.023
Gender, (M:F)	20:25	30:60	0.209
BMI, kg/m^2^ (mean ± SD)	42.1 ± 4.2	45.8 ± 6.1	0.003
Weight, Kg (mean ± SD)	121.9 ± 21.1	131.5 ± 24.1	0.024
Comorbidity, (*n*, %)	36 (80%)	80 (88.9%)	0.163
OSAS, (*n*, %)	8 (17.8%)	33 (36.6%)	0.025
Hypertension, (*n*, %)	18 (40%)	36 (40%)	1
Type 2 diabetes mellitus, (*n*, %)	10 (22.2%)	29 (32.2%)	0.228
NAFLD, (*n*, %)	25 (55.6%)	53 (58.9%)	0.712
Previous abdominal surgery			
Laparoscopic, (*n*, %)	6 (13.3%)	23 (25.5%)	0.001
Open, (*n*, %)	13 (28.8%)	6 (6.7%)	
Intra-operative complications (*n*, %)	1 (2.2%)	4 (4.4%)	0.664
Mean docking time (mean ± SD), min	5.6 ± 1.2	5.4 ± 1.1	0.162
Mean console time (mean ± SD), min	131.6 ± 34.8	151.7 ± 32.4	0.001
Mean total operative time (mean ± SD), min	166.9 ± 39.9	186.9 ± 53.1	0.024
Post-operative ICU, (n, %)	1 (2.2%)	4 (4.4%)	0.664
Post-operative hospital stay, days (Median, IQR)	2 (1–2)	2 (2–5)	0.001
Post-operative NRS (mean ± SD)	3.6 ± 1.8	3.5 ± 1.7	0.752
Patients with early complications, (*n*, %)	3 (6.7%)	6 (6.7%)	1
Readmission, (*n*, %)	2 (4.4%)	1 (1.1%)	0.257

*RYGB* Roux-en-Y Gastric Bypass; *RAS* robot-assisted surgery; *SS* surgical systems; *SD* standard deviation; *IQR* 75% interquartile Range; *BMI* body mass index; *OSAS* obstructive sleep apnea syndrome; *NAFLD* non-alcoholic fatty liver disease; *NRS* numeric rating scale

The incidence of intraoperative complications did not significantly differ among the two groups (1 case and 4 cases in the Hugo^™^-RAS group and in the DaVinci^®^-SS group, respectively) (*p* = 0.664). We reported one case of minimal liver surface injury in the in the Hugo^™^-RAS group; differently, one case of gastro-jejunal microleak detected by blue methylene test, requiring suture stick, and 3 cases of minimal liver surface injury were reported in the DaVinci^®^-SS group. A significant difference was observed in mean console time, with longer required time for the DaVinci^®^-SS group (151.7 ± 32.4 min) compared to the Hugo^™^-RAS group (131.6 ± 34.8 min) (*p* = 0.001). Likewise, the mean OT was significantly greater for the DaVinci^®^-SS group (186.9 ± 53.1 min vs 166.9 ± 39.9 min in the Hugo^™^-RAS group, *p* = 0.024). There were no recorded conversions either from RA approach to laparoscopic approach and from RA approach to open approach in both groups. Early postoperative complications rate was comparable between the two groups (6.7%). We reported an overall inferior POS in the Hugo^™^-RAS group; in detail, despite the same median value (2 days), the IQR in the DaVinci^®^-SS group was higher (2–5) (*p* = 0.001). Readmission rates were comparable: 2 cases in the Hugo^™^-RAS group compared to one case in the DaVinci^®^-SS group (*p* = 0.257). In the DaVinci^®^-SS group, six early complications were reported, consisting of 3 gastro-jejunal anastomotic leaks (2 Clavien–Dindo Grade II and one Clavien–Dindo Grade IIIb), 2 intraluminal gastric bleedings (one Clavien–Dindo Grade I and one Clavien–Dindo Grade IIIb) and one gastro-jejunal anastomosis stenosis (Clavien–Dindo Grade IIIb). Conversely, in the Hugo^™^-RAS-group 2 gastro-jejunal anastomosis leaks occurred (Clavien–Dindo Grade IIIb) and one patient required readmission and antibiotic therapy due to pneumonia (Clavien-Dindo Grade II). Thirty-day mortality rate was nil in both groups.

Table 2 provides a comparative analysis of clinicopathologic characteristics, intraoperative and postoperative results following application of PSM on the two groups, each one including 45 patients. After PSM all operations in the DaVinci^®^-SS group were accomplished with Xi platform. Age, sex, pre-operative BMI, comorbidities and previous abdominal surgeries were comparable between the groups. The reported data show no notable variations regarding the number of intraoperative complications (one in the Hugo^™^-RAS group vs 2 in the DaVinci^®^-SS group, *p* = 1). The docking and console times were comparable (5.4 ± 0.5 min and 144.4 ± 46.9 min for the DaVinci^®^-SS group compared to 5.6 ± 1.2 min and 131.6 ± 34.8 min for Hugo^™^-RAS) (*p* = 0.176, *p* = 0.678). The total OTs were also similar, with Hugo^™^-RAS at 166.9 ± 39.9 min and DaVinci^®^-SS at 179.8 ± 47.1 min (*p* = 0.229). Intraoperative complications rate was comparable (4.4% vs 2.2%, *p* = 1, DaVinci^®^-SS group and Hugo^™^-RAS group, respectively). More in details, we experienced one case of minimal liver surface injury in the Hugo^™^-RAS group; on the other hand, one case of minimal liver surface injury and one case gastro-jejunal microleak detected by blue methylene test, requiring suture of the defect, were reported in the DaVinci^®^-SS group. No conversion was reported. Early complication rates were comparable (*p* = 1). Delving deeper, DaVinci^®^-SS reported one case of intraluminal gastric bleeding (Clavien-Dindo Grade IIIb), one case of gastro-jejunal stenosis (Clavien-Dindo Grade IIIb) and one case of gastro-jejunal anastomotic leak (Clavien-Dindo Grade II). In the Hugo^™^-RAS group 2 cases of gastro-jejunal anastomotic leak (Clavien-Dindo Grade IIIb) and one case of pneumonia, that required readmission and resolved with antibiotic therapy (Clavien-Dindo Grade II), were reported. Summarizing, the rates of gastro-jejunal anastomotic leaks were comparable: 2.2% vs 4.4% (*p* = 1) for DaVinci®-SS group and Hugo^™^-RAS group, respectively. Similarly, there were no noteworthy differences detected in relation to gastro-jejunal stenosis (2.2% vs 0%, *p* = 1, for DaVinci^®^-SS group and Hugo^™^-RAS group, respectively) and intraluminal gastric bleeding (2.2% vs 0%, *p* = 1, for DaVinci^®^-SS group and Hugo^™^-RAS group, respectively). Moreover, the reoperation rate was comparable (4.4% vs 4.4%, *p* = 1, for DaVinci^®^-SS group and Hugo^™^-RAS group, respectively). Postoperative complications are described in detail in the supplementary materials section. Median POS was 2 days for both groups (*p* = 0.052). Readmissions rates were similar in both groups (*p* = 1).

**Table 2:** Clinicopathologic characteristics, intraoperative and postoperative outcomes of Hugo^™^-RAS RYGB (*n* = 45) vs DaVinci^®^-SS RYGB (*n* = 45) after propensity matching score analysis

Variable	Hugo^™^-RAS *N* = 45	DaVinci^®^-SS *N* = 45	p-value
Age, years (mean ± SD)	48.1 ± 10.8	46.3 ± 10.1	0.416
Gender, (M:F)	20:25	18:27	0.671
BMI, kg/m^2^ (mean ± SD)	42.1 ± 4.2	43.5 ± 5.2	0.163
Weight, Kg (mean ± SD)	121.9 ± 21.1	126.7 ± 23.2	0.307
Comorbidity, (*n*, %)	36 (80%)	30 (66.7%)	0.155
OSAS, (*n*, %)	8 (17.8%)	6 (13.3%)	0.563
Hypertension, (*n*, %)	18 (40%)	22 (48.9%)	0.398
Type 2 Diabetes Mellitus, (*n*, %)	10 (22.2%)	13 (28.9%)	0.509
NAFLD, (*n*, %)	25 (55.6%)	24 (53.3%)	0.833
Previous abdominal surgery			
Laparoscopic, (*n*, %)	6 (3.3%)	13 (28.9%)	0.075
Open, (*n*, %)	13 (28.9%)	6 (3.3%)	
Intra-operative complications (*n*, %)	1 (2.2%)	2 (4.4%)	1
Mean docking time (mean ± SD), min	5.6 ± 1.2	5.4 ± 0.5	0.176
Mean console time (mean ± SD), min	131.6 ± 34.8	144.4 ± 46.9	0.678
Mean total operative time (mean ± SD), min	166.9 ± 39.9	179.8 ± 47.1	0.229
Post-operative ICU, (*n*, %)	1 (2.2%)	2 (4.4%)	1
Post-operative hospital stay, days (Median, IQR)	2 (1–2)	2 (2–2)	0.052
Post-operative NRS (mean ± SD)	3.6 ± 1.8	3.6 ± 1.6	1
Patients with early complications, (*n*, %)	3 (6.7%)	3 (6.7%)	1
Readmission, (*n*, %)	2 (4.4%)	1 (2.2%)	1

*RYGB* Roux-en-Y Gastric Bypass; *RAS* robotic-assisted surgery; *SS* surgical systems; *SD* standard deviation; *IQR* 75% interquartile Range; *BMI* body mass index; *OSAS* obstructive sleep apnea syndrome; *NRS * numeric rating scale

## Discussion

This retrospective cohort study shows that complications rate and OTs of RYGB performed by means of the Hugo^™^-RAS platform were comparable to the well-established and widely used DaVinci^®^-SS system.

The potential advantages of RA surgery encompass enhanced ergonomics, three-dimensional vision, tremor reduction and the use of devices with increased degrees of freedom [3]. These advantages may lead to improved surgical skills and optimized efficiency when compared to traditional laparoscopic surgery [7]. Indeed, the benefit of robotic platforms relies on a more accurate dissection and easier intracorporeal anastomoses construction with lower surgeon’s physical stress [25]. Recent evidence suggests that surgeons are increasingly utilizing and incorporating robotic platforms in routine activities [26]. As stated in the 7^th^ IFSO global registry report, up to 10.9% of primary bariatric operations and 12.4% of reoperative procedures were accomplished with RA approach in 2020 and 2021, respectively [3]. Despite these advantages, the broader adoption of RA surgery has been hindered by increased costs, longer OTs and limited platform accessibility [7]. On the other hand, the limited availability of robotic platforms probably hampers the execution of the required number of robotic procedures for the learning curve’s plateau that would significantly reduce OT and probably mitigate the high costs.

Despite systematic reviews and meta-analyses have not displayed any evidence regarding enhanced clinical outcomes with RA bariatric operations compared to laparoscopic ones so far [27–29], some retrospective studies showed potential advantages in terms of conversion rate and complications. Such benefit is reported to be particularly relevant in demanding cases, including patients with class IV obesity, reoperative operation, RYGB and hypoabsorptive/malabsorptive procedures such as SADI-S [11, 12, 26]. Due to the result of economic concerns and limited availability of robotic platforms in clinical practice, RA surgery has been primarily addressed to these patients’ category [11, 12].

The application of the DaVinci^®^-SS platform in clinical practice since 2013 allowed us to acquire the necessary learning curve for robotic surgery. Thereafter, viability of Hugo^™^-RAS has been assessed following our initial documented application to adrenalectomy, detailing the platform's configurations and applications [19]. Subsequently, we validated the feasibility of Hugo^™^-RAS RYGB, reporting the outcomes of the first 15 cases of this surgical procedure performed by means of such a platform [21].

Undeniably, any new technology introduced in clinical practice must be compared with the current validated standard of care. To date, recent literature reports have introduced comparison of the new Hugo^™^-RAS platform with DaVinci^®^-SS mainly in the fields of urological and gynecological surgery. These studies showed no substantial disparities between the two systems with respect to operative, postoperative and functional or oncologic outcomes [30–32]. Specifically, in urological surgery, the platforms’ comparison was mainly applied to procedures such as prostatectomies and sacrocolpopexy for pelvic organs prolapse treatment [30–32]. The findings indicate that both platforms are related to comparable surgical precision, OTs and recovery times. Similarly, in gynecological surgery, where robotic assistance is often utilized for complex hysterectomies, the Hugo^™^-RAS platform has demonstrated equivalent effectiveness in achieving successful surgical outcomes and comparable complications rates [30–32].

Despite the promising results in other surgical fields, comparative studies regarding the application of the two robotic platforms to bariatric surgery are still lacking.

Regarding the main outcome of this investigation, our results showed similar postoperative early complications, with a 6.7% rate in both groups. Moreover, our results are also consistent with the reported data from a recent meta-analysis [26] analyzing 28 studies with a total of 82,155 patients, including 9051 RA-RYGBs and 73,104 laparoscopic RYGBs. In the RA surgery group, the overall complications rate was 6.7%, comparably with our experience. Other authors described different results, from the 0% of Sanchez et al. [33] to the 33.1% of Renaud et al. [34]. Delving deeper, in our experience the rates of gastro-jejunal anastomotic leaks were comparable: 2.2% vs 4.4% (*p* = 1) for DaVinci^®^-SS group and Hugo^™^-RAS group, respectively. These data are in line with Fantola et al. [35] results, with an anastomotic leak rate of 4.6% over more than 300 RA RYBGs. Moreover, both groups exhibited a reoperation rate of 4.4% for the management of Clavien–Dindo IIIb complications, consisting of hemorrhage, gastro-jejunal anastomotic leaks and gastro-jejunal anastomotic stenosis, which occurred within 30 days from the index operation. Our reoperative treatment rates are also in line with Leang et al. [26] and even inferior compared to those ones reported in other studies (10.2%) [35]. Similarly, we encountered only one (2.2%) gastro-jejunal stenosis, which occurred in the DaVinci^®^-SS group. Leang et al. [26] reported a 2.3% rate of strictures after robotic RYGB in 7 out 28 analyzed trials. However, other authors reported stenosis rate in up to 7% of cases [36].

We experienced similar intraoperative complications rate (2 patients vs 1 patient, *p* = 1, in DaVinci^®^-SS group and Hugo^™^-RAS group, respectively) compared to the widely heterogeneous experiences reported in the literature, ranging from 0% [37, 38] to 11% [39].

About the secondary outcomes of our study, OTs are in line with literature reports. Delving deeper, the mean OT was 178.9 ± 47.1 min and 166.9 ± 39.9 min for the DaVinci^®^-SS group and the Hugo^™^-RAS group, respectively, while among the 19 studies analyzed in Leang et al. [26] meta-analysis, OTs vary between 108 and 353 min. Notably, despite the differences in docking realization among the two robotic platforms (DaVinci^®^-SS vs Hugo^™^-RAS) [7, 20], we reported comparable OTs results (5.4 ± 0.5 min vs 5.6 ± 1.2 min, *p* = 0.176, for DaVinci^®^-SS and Hugo^™^-RAS, respectively). Nonetheless, Beckmann et al. [40] carried comparable mean docking time value for DaVinci^®^-SS bariatric operations. Similarly, median POS (2 days for both groups, *p* = 0.052) is also comparable with results from 20 trials (ranging between 1.7 and 10.5 days) [26].

Hospital readmission involved only one patient from the DaVinci^®^-SS group after the initial discharge following surgery. Comparably, two patients from the Hugo^™^-RAS group required readmission. Similar readmission rate (2.9%) has been reported in the meta-analysis of Leang et al. [26] analyzing 11 out 28 studies.

This study is notable for being the first to compare DaVinci^®^-SS and Hugo^™^-RAS platforms for RA RYGB. Furthermore, we presented a monocentric series from a national referral center for bariatric surgery. The reported data are related to surgical operations performed in a high-volume single institution, thus limiting the general validity of the findings. Indeed, these results might not be applicable to other settings with different patients’ populations, surgical protocols and healthcare resources. Additionally, the follow-up length may not be sufficient to evaluate all relevant long-term complications and outcomes. Indeed, long-term follow-up after bariatric operations is crucial to fully understand the procedure impact on clinical outcome.

Although use of PSM represents a useful means to control possible confounding variables, unmeasured factors which still could potentially influence the outcomes remain, including variations in postoperative care or patient-specific characteristics. However, the homogeneity in patients’ selection after PSM is also related to the use of a single type (Xi) platform in the DaVinci^®^-SS group. Moreover, the surgical steps of the RYGB were the same regardless the different robotic platforms. In detail, all gastro-jejunal and jejuno-jejunal anastomoses were accomplished with the same technique and all procedures were performed by the means of laparoscopic stapler devices. Indeed, the robotic stapler for DaVinci^®^-SS procedures was introduced in January 2024, after the study period.

Moreover, although no unique precise parameter is currently available to describe the complexity of bariatric patients, in an effort to mitigate this potential bias, we conducted an analysis of patients based not only on BMI, age, and sex but also on NAFLD.

On the other hand, this study also presents several limitations. Firstly, its retrospective nature with a relatively limited sample size represents one of the main limits. Secondly, although all operations have been conducted by a senior minimally invasive bariatric surgeon after acquiring a preliminary learning curve of at least 200 laparoscopic RYGB before DaVinci^®^-SS RA surgeries and all Hugo^™^-RAS-RYGBs were performed after completing a learning curve of at least 30 DaVinci^®^-SS-bariatric operations, the inclusion of the preliminary experience in our series may represent a bias for the study. Furthermore, even though cost analysis does not represent an endpoint of our study, it is to highlight that the introduction of a new competitor in any market typically leads to a modulation of the supply and a subsequent potential reduction in costs [41]. As a result of competition policy, encouraging entrepreneurial spirit and efficiency thus helping to lower prices while improving quality, the cost of robotic systems should gradually reduce, making robotic bariatric surgery more cost-effective [42]. Moreover, the cost comparison of DaVinci^®^-SS and Hugo^™^-RAS robotic systems is also influenced by several external factors. Indeed, the robotic systems-related costs, including purchase, maintenance, training and consumables, can widely vary among different healthcare systems and over time. Therefore, given all the aforementioned considerations, drawing definitive conclusions about their cost-effectiveness is still challenging.

Even if the cost-analysis has not been considered as outcome of the present study, it is to highlight that in our center, even though an established bariatric robotic program has been assessed from 2013 with the DaVinci^®^-SS platform, no more than 2% (approximately 100 cases) of all bariatric surgical interventions were planned using RA technique since 2022. However, this trend was reversed over the past year. Indeed, with the introduction of Hugo^™^-RAS platform and the subsequent more competitive economic accessibility, RA to bariatric surgery has been more broadly applicable, with the resulting performed 97 cases during 2023 (Fig. 2). This accounted for 14.7% of all bariatric procedures and approximately 30% of all RYGBs performed during 2023.

Lastly, further critical outcomes, such as patient satisfaction, quality of life and long-term weight loss, are not addressed. These additional factors may significantly impact on the overall success and acceptability of the surgical procedures and their absence provides a limited view of the comparative effectiveness of the robotic systems. Recognizing these limitations allows for a more balanced interpretation of the study's results and highlights the need for further research to comprehensively address these issues.

In this comparative analysis some considerations have to be taken into account. The two robotic platforms confronted each other at separate moments in their respective stages of “evolution”. To be more precise, the first DaVinci^®^-SS platform was established in 1999, whereas the Hugo^™^-RAS was developed only two years ago. Over the past two decades, the DaVinci^®^-SS platform has undergone significant improvement. Contrarily, the Hugo^™^-RAS platform is now in the advancement and refining phase. Consequently, the Hugo^™^-RAS robotic platform currently has a restricted selection of devices [19, 20].

In conclusion, our study compares clinical outcome after RYGB performed using Hugo^™^-RAS and DaVinci^®^-SS robotic platforms, indicating that both systems are related to comparable safety profiles.

Although DaVinci^®^-SS still remains the widely adopted platform in clinical practice, likely due to its longer track record and established efficacy worldwide, this study underscores the potential efficiency of the Hugo^™^-RAS in the armamentarium of bariatric surgeons to perform RA operations.

However, further research, including larger studies and randomized clinical trials, is necessary to corroborate these preliminary conclusions and investigate the long-term benefits and cost-effectiveness of the Hugo^™^-RAS platform.

## Supplementary Information

Below is the link to the electronic supplementary material.

Supplementary file1 (DOCX 18 KB)

## Data Availability

No datasets were generated or analysed during the current study.
