# Supplementary material for: C omparison between DaVinci ® and Hugo ™ -RAS Roux-en-Y Gastric Bypass in bariatric surgery
Source: J Robot Surg. 2024 Aug 6;18(1):303. doi: 10.1007/s11701-024-02063-w (PMC11303586; doi:10.1007/s11701-024-02063-w)
Supplement: Supplementary file 1 — Supplementary file1 (DOCX 18 KB) [file 11701_2024_2063_MOESM1_ESM.docx]

**Supplementary materials: report of the postoperative complications in the selected population after propensity score matching analysis.**

1. On POD 1 after DaVinci-RSS RYGB, a 34-years-old male patient (46.4 kg/m²BMI) was readmitted to ICU due to hemorrhagic shock. Exploratory laparoscopy and concurrent esophagogastroscopy were promptly performed, showing a penetrating ulcer at the gastro-jejunal anastomosis site as cause of the complication. During the procedure, hemostasis was achieved by means of positioning of two endoclips (Clavien-Dindo Grade IIIb complication). Despite initial clinical and biochemical stabilization, on POD 27 the patient's postoperative course was complicated by episodes of hematemesis and melena, leading to a second episode of hemorrhagic shock. A further urgent endoscopic intervention was performed, with placement of additional endoclips to ensure hemostasis. After the re-intervention the patient presented further recurrent multiple episodes of melena, though in absence of alteration of vital parameters or significant modification of general clinical conditions. With the aim to achieve definitive patient’s treatment, on POD 31 a surgical revision of the gastro-jejunal anastomosis was realized. Ultimately, the patient was discharged in stable clinical conditions on POD 38.
2. A 30-years-old male patient with a preoperative BMI of 44.1 kg/m² underwent a Hugo-RAS RYGB. The postoperative course was characterized by fever and abdominal pain. On POD 7 an abdominal CT scan with endovenous and water-soluble contrast revealed a gastro-jejunal fistula, requiring surgical treatment. Suturing of the anastomotic dehiscence and peritoneal cavity toilette were performed (Clavien-Dindo Grade IIIb complication). Despite the urgent intervention, on POD 14 patient’s impaired clinical and biochemical conditions led to the repetition of imaging study, showing the persistence of the complication. Endoscopic management was chosen, with the placement of two Spaxus endoprosthesis. The patient was discharged in stable condition on POD 43.
3. Sixty-one-years-old male patient (48 kg/m² preoperative BMI) undergone Hugo-RAS RYGB. On POD 2, the patient referred to the emergency department with fever (38.3°C). An abdominal CT scan was performed, showing no pathologic reports. However, basal thoracic scans evidenced radiologic reports compatible with pneumonia. The complication management only required diagnostic interventions and medical treatment (Clavien-Dindo Grade II complication). The patient was discharged 2 days after.
4. Fifty-two-years-old patient with a preoperative BMI of 57.3 kg/m² who underwent a DaVinci-RSS RYGB. On POD 2, routine upper gastrointestinal contrast study with water-soluble contrast revealed a micro leak at the gastroenteric anastomosis. A conservative management approach was opted, including empirical intravenous antibiotics and antifungal medications to prevent potential infections and parenteral nutritional support (Clavien-Dindo Grade II). On POD 7 radiological examination did not detect any further evidence of leakage. The patient was discharged in good clinical conditions on POD 9.
5. The fifty case is a 54-years-old male patient with a preoperative BMI of 35.9 kg/m², who underwent Hugo-RAS RYGB. Routine upper gastrointestinal contrast study with water-soluble contrast performed on POD1 revealed contrast leak along the left side of the gastric pouch suspicious for a dehiscence. Therefore, an urgent laparoscopic procedure was performed, consisting in draining of a subdiaphragmatic collection, suturing of the gastric pouch dehiscence at the cardias and a fundectomy (Clavien-Dindo Grade IIIb complication). On POD 12, enteric-like material was observed in the abdominal drain. An abdominal CT scan was promptly performed, showing a collection near the distal esophagus and a right pleural effusion. The patient underwent a further revisional surgical treatment, consisting in excluding cervical esophagostomy, laparoscopic drainage, proximal exclusion of the efferent loop and a laparoscopic Vitzel jejunostomy. On POD 52, intestinal recanalization with entero-enteric anastomosis was successfully performed. Upper gastrointestinal contrast study showed no contrast leakage, confirming the adequate surgical outcome. On POD 114 closure of cervical esophagostomy was planned.
6. A 43-year-old patient with a preoperative BMI of 41.6 kg/m² underwent a DaVinci-RSS RYGB. On POD 1, the patient experienced significant vomiting and abdominal pain. An upper gastrointestinal contrast study with water-soluble contrast was performed, indicating the gastro-jejunal anastomosis stenosis (Clavien-Dindo Grade IIIb complication). A laparoscopic revision of the gastro-jejunal anastomosis was performed, with successful resolution of the obstruction. The subsequent patient’s postoperative course was regular with no further complications. The patient resumed oral intake shortly after and was monitored closely for any symptoms’ recurrence. On POD 5, the patient was discharged in good clinical condition.
